# The Transcriptional Corepressor RIP140 Regulates Oxidative Metabolism in Skeletal Muscle

**DOI:** 10.1016/j.cmet.2007.08.004

**Published:** 2007-09-05

**Authors:** Asha Seth, Jennifer H. Steel, Donna Nichol, Victoria Pocock, Mande K. Kumaran, Asmaa Fritah, Margaret Mobberley, Timothy A. Ryder, Anthea Rowlerson, James Scott, Matti Poutanen, Roger White, Malcolm Parker

**Affiliations:** 1 Institute of Reproductive and Developmental Biology, Imperial College London, Du Cane Rd, London W12 ONN, UK; 2 School of Biomedical and Health Sciences, King's College London, London SE1 1UL, UK; 3 MRC Clinical Sciences Centre, Faculty of Medicine, Imperial College London, London W12 ONN, UK; 4 Department of Histopathology, Charing Cross Hospital, Fulham Palace Road, London W6 8RF, UK; 5 Imperial College London, South Kensington, London SW7 2AZ, UK; 6 Department of Physiology, Institute of Biomedicine, University of Turku, Kiinamyllynkatu 10, 20520 Turku, Finland

**Keywords:** DNA

## Abstract

Nuclear receptor signaling plays an important role in energy metabolism. In this study we demonstrate that the nuclear receptor corepressor RIP140 is a key regulator of metabolism in skeletal muscle. RIP140 is expressed in a fiber type-specific manner, and manipulation of its levels in null, heterozygous, and transgenic mice demonstrate that low levels promote while increased expression suppresses the formation of oxidative fibers. Expression profiling reveals global changes in the expression of genes implicated in both myofiber phenotype and metabolic functions. Genes involved in fatty-acid oxidation, oxidative phosphorylation, and mitochondrial biogenesis are upregulated in the absence of RIP140. Analysis of cultured myofibers demonstrates that the changes in expression are intrinsic to muscle cells and that nuclear receptor-regulated genes are direct targets for repression by RIP140. Therefore RIP140 is an important signaling factor in the regulation of skeletal muscle function and physiology.

## Introduction

Skeletal muscles required for sustained contractile activity such as the soleus contain mainly “slow-twitch” oxidative fibers rich in mitochondria, while those involved in rapid, shorter bursts of activity such as the gastrocnemius and extensor digitorum longus (edl) contain more “fast-twitch” fibers rich in glycolytic enzymes for anaerobic metabolism. Slow fibers tend to express type I myosin heavy chains (MyHC) while fast fibers express type IIA and IIB MyHC (Bassel-Duby and Olson, 2006; Schiaffino and Reggiani, 1996). Skeletal muscle may also contain a fourth type of fiber, IIX, that exhibits fast-twitch properties intermediate between IIA and IIB fibers but that may be oxidative like type I fibers (Larsson et al., 1991). Fiber-type composition can be modulated by a number of different factors such as exercise, aging, or hormonal changes (Wu et al., 2003). Exercise tends to shift MyHC expression and fiber-type properties following the pathway IIB→IIX→IIA→I (Baldwin and Haddad, 2001). Mechanistically, altered motor neuronal activity leading to changes in intracellular calcium concentration is thought to underlie these changes (Chin et al., 1998; Olson and Williams, 2000), and although a number of transcriptional regulators that are able to remodel skeletal muscle have been identified (Lin et al., 2002; McCullagh et al., 2004), it is still uncertain how the signaling pathways are coordinated.

Nuclear receptors and their associated cofactors are emerging as important regulators of transcriptional control of energy metabolism and fiber types in skeletal muscle. Targeted expression of an activated form of peroxisome proliferator-activated receptor δ (PPARδ) in skeletal muscle promotes fast to slow muscle transformation and increases the expression of muscle genes involved in fatty-acid oxidation, mitochondrial respiration, and slow-twitch contractile apparatus (Wang et al., 2004). Similarly overexpression of the orphan nuclear receptor estrogen-related receptor α (ERRα) in C2C12 myotubes results in the upregulation of a subset of genes involving the same metabolic pathways, although an effect in vivo on fiber-type switching has not been analyzed (Huss et al., 2004). Both PPARδ and ERRα can be activated by PPAR coactivators, (Kamei et al, 2003). The expression of PGC-1α is higher in type I oxidative muscles such as the soleus compared with fast-twitch muscles such as edl and has been demonstrated to activate mitochondrial biogenesis and oxidative metabolism (Wu et al., 1999). Moreover, overexpression of this cofactor in muscle leads to the conversion of fast to slow fibers accompanied by increased expression of metabolic genes (Lin et al., 2002). PGC-1β is also expressed in the soleus muscle, but the highest levels are found in the edl and overexpression has recently been found to promote the formation of oxidative type IIX fibers (Arany et al., 2007). These data indicate that the metabolic properties of skeletal muscle can be regulated by nuclear receptors with coactivators playing a key role.

In contrast to transcriptional activation, the contribution of transcriptional repression by nuclear receptors in muscle physiology is poorly understood. We have studied the role of the nuclear receptor corepressor RIP140 (Cavailles et al., 1995; L'Horset et al., 1996), which regulates fat storage in adipose tissue (Leonardsson et al., 2004) at least in part by repressing the expression of metabolic genes (Powelka et al., 2006). In this study we demonstrate in genetically manipulated mice that RIP140 plays an important role in skeletal muscle regulating gene networks that determine mitochondrial activity and fiber type. Analysis of differentiated myofibers in culture demonstrates the recruitment of RIP140 to the promoters of target genes that are subject to regulation by PPARδ and ERRα. Thus we conclude that RIP140 is required for the coordinated control of glucose and fat metabolism and plays an important role in skeletal muscle physiology.

## Results

### Reduction in RIP140 Expression Increases Oxidative Fibers in Skeletal Muscle

**Figure 1:**
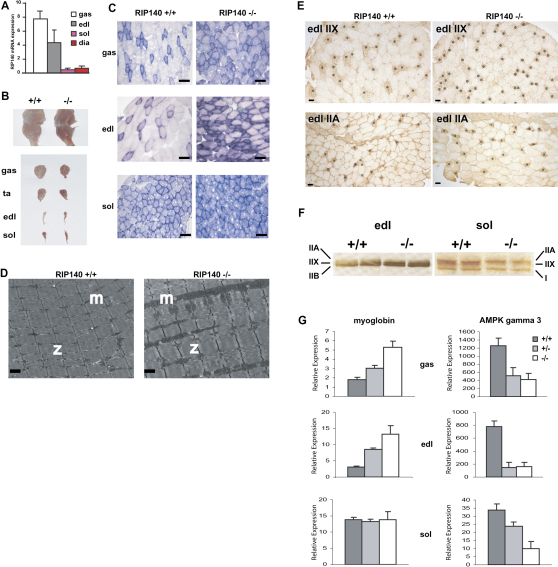
Decreased RIP140 Expression Increases the Proportion of Oxidative Type I Fibers in Skeletal Muscle and Expression of Myosin Isoforms and Fiber-Type Markers (A) Real-time PCR analysis of RIP140 mRNA expression in type I and II muscle groups. Type II: gas, gastrocnemius; edl, extensor digitorum longus. Type I: sol, soleus; dia, diaphragm (n = 4). Data are expressed as mean ± standard error of the mean (SEM). (B) Morphology of muscle from wild-type (WT) and RIP140 null mice. gas, gastrocnemius; edl, extensor digitorum longus; ta, tibalis anterior; sol, soleus. (C) Histochemical staining for succinate dehydrogenase in muscle sections from WT and RIP140 null mice taken from the indicated muscle groups (scale bars = 100 μm). (D) Transmission electron microscope analysis of edl muscle from WT (RIP140^+/+^) and null (RIP140^−/−^) mice (×5600 magnification). m, mitochondria; z, z line (scale bars = 2 μm). (E) Analysis of myosin IIX and IIA expression in edl of WT and RIP140 null mice. Asterisks in upper panels indicate IIX-positive fibers identified with 6H1 type IIX-specific antibody. Asterisks in lower panels indicate position of IIA-positive fibers. (F) Analysis of edl and soleus muscle protein extracts by SDS-PAGE and silver staining. (G) Real-time PCR analysis of fiber-type markers myoglobin and AMPγ3 expression in WT, heterozygous, and null mice in indicated muscle groups (n = 3–5). Data are expressed as mean ± SEM.

Analysis of skeletal muscle for RIP140 mRNA expression indicated that the corepressor is relatively highly expressed in gastrocnemius and edl muscles that are rich in glycolytic fast-twitch fibers, while in contrast expression is low in soleus and diaphragm that are rich in oxidative slow-twitch fibers (Figure 1A). The deletion of the RIP140 gene leads to a change in appearance with the gastrocnemius, tibalis anterior, and edl muscles becoming redder than controls (Figure 1B). To investigate these differences we determined oxidative capacity by analyzing mitochondrial activity. Histochemical measurement of succinate dehydrogenase (SDH) activity demonstrates increased intensity of staining in the gastrocnemius and the edl in the absence of RIP140 but minimal changes in the soleus, which already exhibits appreciable SDH activity (Figure 1C). RIP140 heterozygous mice express reduced levels of RIP140 in the gastrocnemius and in the edl (data not shown), resulting in intermediate levels of mitochondrial activity as judged by SDH staining (Figure S1 available with this article online).

Increased SDH activity observed in RIP140 null mice indicates that loss of RIP140 leads to either an increase in the total number of mitochondria, the activity of the mitochondria, or both. Transmission electron microscopy of the edl indicates an increase in both size and quantity of mitochondria in null mice in comparison to normal controls (Figure 1D) with intermediate values for heterozygous mice (Figure S2A). Quantitation of the mitochondrial encoded gene *cox II* normalized to nuclear DNA (Figure S2B) indicated that mitochondrial DNA increased 2-fold in the absence of RIP140.

To determine fiber-type composition, metachromatic ATPase and MyHC staining of muscle sections derived from wild-type (WT) and RIP140 null mice was analyzed (Figure S3 and data not shown). By immunostaining with specific antisera (Lucas et al., 2000) the relative proportions of type I, IIA, IIX, and IIB fibers in the soleus and edl muscles were determined in the presence and absence of RIP140. Analysis of serial sections of edl demonstrated an increase in the proportion of fibers expressing myosins IIA and IIX and a corresponding reduction in myosin IIB (Figure 1E and Table S1) consistent with the increase in oxidative capacity of this muscle. In the soleus, there was a small increase in the proportion of type 1 fibers at the expense of type IIA and IIX fibers although all three types of fiber are predominantly oxidative (Table S1).

To investigate the effect of RIP140 on myosin expression, protein extracts from edl and soleus were analyzed for specific isoforms by SDS-PAGE and silver staining. The edl contains predominantly MyHC IIB and the soleus contains MyHC I, IIA, and IIX (Figure 1F). In RIP140 null mice myosin IIX increases in the edl muscle while in the soleus the level of IIX is reduced with minimal changes in the other myosin proteins. We conclude that RIP140 suppresses the formation of oxidative fibers in the edl muscle, given the SDH staining results, with a modest effect on myosin gene expression. In addition to myosin levels we also examined expression of myoglobin, an oxygen transporter responsible for the red coloration of oxidative fibers, and AMPKγ3, an isoform predominantly expressed in glycolytic skeletal muscle. In gastrocnemius and edl, the expression of myoglobin showed a dose-dependent increase with decreasing expression of RIP140 (Figure 1G). Conversely the expression of AMPKγ3, expressed in glycolytic fibers, showed a pronounced decrease with the removal of RIP140. In soleus, the absence of RIP140 had no effect on myoglobin expression, consistent with the lack of change in SDH staining; however, the expression of AMPKγ3 decreased, suggesting reduced glycolytic activity and indicating that the low levels of RIP140 expressed in this muscle group are still functionally significant.

### Overexpression of RIP140 Reduces Oxidative Activity in Skeletal Muscle

**Figure 2:**
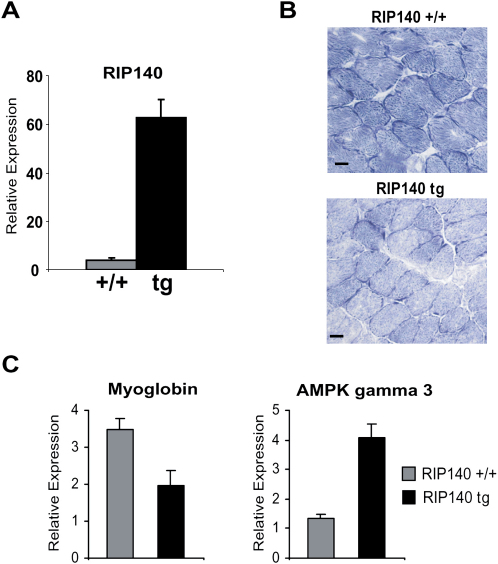
Exogenous Expression of RIP140 Reduces the Proportion of Oxidative Fibers in Skeletal Muscle (A). Expression of endogenous RIP140 and RIP140 transgene in soleus muscle (n = 7). Data are expressed as mean ± SEM. (B) Histochemical staining for succinate dehydrogenase in muscle sections from WT and RIP140 Tg mice taken from soleus muscle (scale bars = 20 μm). (C) Real-time PCR analysis of mRNA expression of fiber-type markers in WT and transgenic (RIP140 Tg) mice soleus muscle (n = 7). Data are expressed as mean ± SEM.

The effect of overexpression of RIP140 in transgenic mice was examined focusing on the soleus muscle, where endogenous RIP140 levels are low and a putative repressive action may be most clearly observed. The expression of RIP140 was about 15-fold higher than endogenous levels, similar to those normally found in the edl muscle (Figure 2A). This results in a decrease in the intensity of SDH staining in comparison to controls (Figure 2B), consistent with a reduction in oxidative activity. In addition, myoglobin expression was decreased whereas AMPKγ3 expression was increased, suggesting an increase in glycolytic activity (Figure 2C). Thus the expression level of RIP140 is a key determinant of the relative oxidative activity exhibited by different types of muscle types.

To investigate whether RIP140 may act to repress the transformation of fibers with increased oxidative activity that occurs during physical activity we investigated the effects of exercise on the tibalis anterior muscle. Following 6 weeks of voluntary exercise in WT mice, oxidative activity was increased and the proportion of IIA fibers was increased by 10% with a corresponding decrease in IIB fibers (Figure S4). This exercise-induced transformation was maintained in the RIP140 transgenic mice with an increase in oxidative activity and a 17% increase in the proportion of IIA fibers at the expense of IIX and IIB fibers, both of which declined by approximately 8% (Figure S4). Thus we conclude that RIP140 does not prevent the formation of oxidative fibers during exercise but is a key determinant of the relative oxidative activity.

### Absence of RIP140 Enhances Multiple Metabolic Pathways in Skeletal Muscle

**Figure 3:**
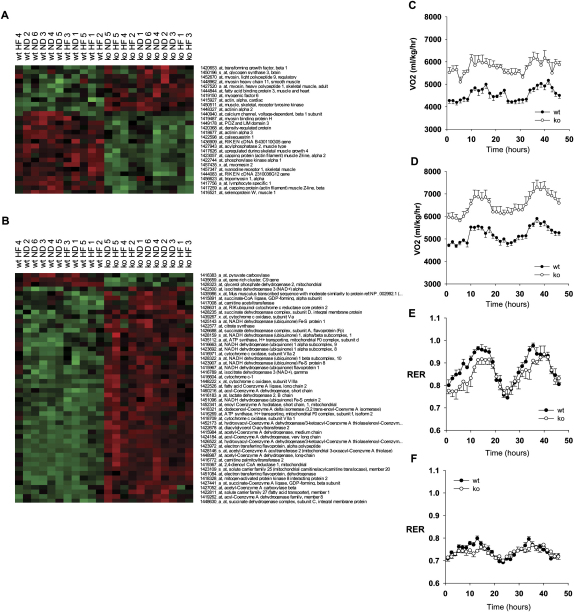
Expression Profiling and Metabolic Studies in WT and RIP140 Null Mice (A) Cluster analysis of genes significantly altered in the null muscle tissue with a significance value of p < 0.01 with a specific defined role in muscle cell development, structure, or function. Red and green colors indicate relative expression levels (red = increased expression, green = reduced expression). (B) Cluster analysis of genes significantly altered in the null muscle tissue with a significance value of p < 0.01 with a specific defined role in metabolic pathways (red = increased expression, green = reduced expression). (C) Forty-eight hour profile of oxygen consumption in WT and RIP140 null (KO) mice (n = 6) maintained on normal diet. Data are expressed as mean ± SEM. (D) Forty-eight hour profile of oxygen consumption in WT and RIP140 null (KO) mice (n = 6) maintained on high-fat diet. Data are expressed as mean ± SEM. (E) Forty-eight hour profile respiratory exchange ratio (RER) in WT and RIP140 null (KO) mice maintained on a normal diet. Data are expressed as mean ± SEM. (F) Forty-eight hour profile RER in WT and RIP140 null (KO) mice maintained on a high-fat diet. Data are expressed as mean ± SEM.

To investigate potential molecular mechanisms leading to the changes observed in muscle physiology in the absence of RIP140, we used Affymetrix microarrays to perform expression profiling on RNA isolated from the gastrocnemius of WT and RIP140 null mice. The number of probe sets that were differentially expressed with a significance p value of 0.05 was 3044, of which 1196 (39%) were upregulated and 1848 (61%) downregulated. (For a list of genes see Table S2.) Inspection of genes with functions in myoblast differentiation, structure, or function indicated that 10 genes were upregulated and 18 downregulated (Figure 3A). The upregulated genes included Fatty Acid-Binding Protein (FABP3), which was increased by exercise in type II muscle (van Breda et al., 1992; Wu et al., 2003). A second gene set consisting of enzymes involved in oxidative phosphorylation, fatty-acid oxidation, TCA cycle, glycolysis, and triglyceride synthesis (Tables S3A, S3B, and S3C) indicates that 47 (94%) of these genes were upregulated whereas only 3 were downregulated (Figure 3B). The asymmetrical effect of RIP140 deletion on metabolic gene expression is dramatically illustrated when hierarchical clustering is applied to the gene set. This pattern of gene regulation contrasts with the heat map generated for the muscle-specific genes that showed slightly more downregulated genes, in line with the global pattern of expression changes. Of the five pathways examined, fatty-acid oxidation was the pathway most affected by RIP140 deletion with 20 of the 27 genes on the list significantly upregulated. Only three genes were downregulated by the loss of RIP140. Two of these genes, pyruvate carboxylase and glycerol phosphate dehydrogenase, encode glycolytic enzymes, reflecting the change observed in fiber type from glycolytic to oxidative. We also examined the effects of high-fat feeding for 3 months since flux through metabolic pathways is primarily regulated by nutrient intake. Interestingly mice lacking RIP140 still showed an upregulation of metabolic genes, including those involved in fatty-acid oxidation (Figure 3B). The clustering indicates that the muscle of WT mice responds to the high-fat diet with a shift in physiology resulting in increased expression of these metabolic genes; nevertheless the expression levels are still less than those observed in null mice on either diet. Overall these results indicate that the corepressor RIP140 is an important regulatory factor in skeletal muscle for the coordinated regulation of many key components in glucose and fat metabolism.

The physiological role of RIP140 was examined by determining the diurnal pattern of O_2_ consumption in mice maintained on either normal chow or high-fat diet. These studies demonstrate that RIP140 null mice have an approximately 25% increased rate of oxygen consumption compared to controls (Figure 3C); furthermore the increased energy expenditure exhibited by the null mice is maintained after a 3 month period of high-fat feeding (Figure 3D), consistent with the maintenance of differential metabolic gene expression. Mice lacking RIP140 exhibit a decreased respiratory exchange ratio (RER) in comparison to WT controls (Figure 3E), indicating that the null mice utilize a greater proportion of fat as an energy substrate in agreement with the upregulation of fatty-acid oxidation genes. As expected, high-fat feeding decreases the RER as a greater proportion of fat is oxidized (Figure 3F) with a similar trend observed in the null mice. Therefore, although a lack of RIP140 leads to an alteration in the metabolic function of skeletal muscle the ability of RIP140 null mice to respond to a dietary challenge is maintained.

### A Subset of the Target Genes of the Nuclear Receptors PPARδ and ERRα Are Targets for Repression by RIP140 In Vivo

**Figure 4:**
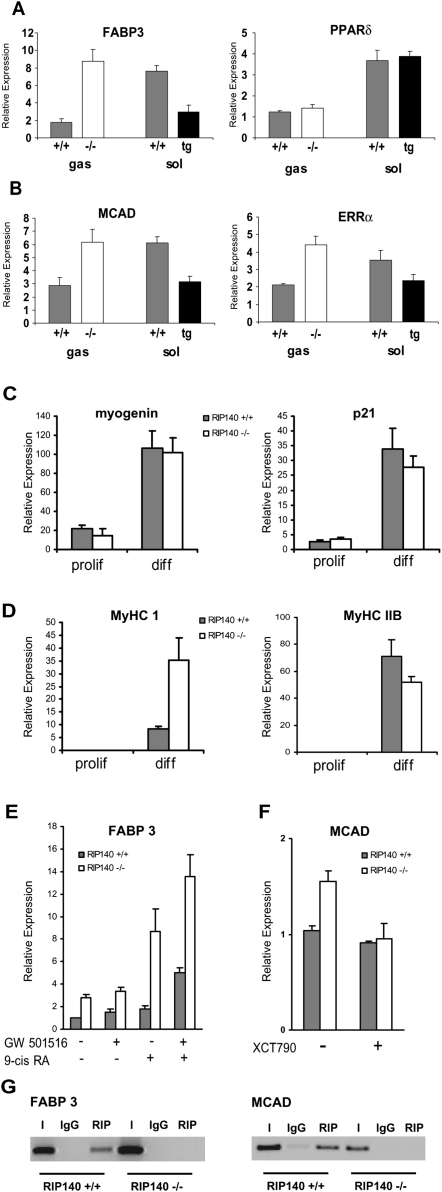
RIP140 Regulates Expression of FABP3 and MCAD in Type I and Type II Muscle and in Differentiated Myoblasts (A) Real-time PCR analysis of PPARδ and target gene FABP3 mRNA expression in gastrocnemius (gas) or soleus (sol) muscle of mice either devoid of or overexpressing RIP140 (n = 4–7). Data are expressed as mean ± SEM. (B) Real-time PCR analysis of ERRα and target gene, MCAD, mRNA expression in gastrocnemius or soleus muscle of mice either devoid of or overexpressing RIP140 (n = 4–7). Data are expressed as mean ± SEM. (C) Real-time PCR analysis of myoblast differentiation markers myogenin and p21 in proliferating WT and RIP140 null myoblasts and differentiated myotubes. Data are expressed as mean ± SEM. (D) Real-time PCR analysis of MyHC isoform expression in proliferating (prolif) WT and RIP140 null myoblasts and differentiated (diff) myotubes. Data are expressed as mean ± SEM. (E) Ligand-dependent repression of FABP3 mRNA expression in WT and RIP140 null myotubes. Myoblasts were differentiated for 4 days as described (Experimental Procedures). Subsequently, the myotubes were treated with agonists for RXR (9-*cis* retinoic acid; 0.1 μM), PPARδ (GW501516; 1 μM), both together, or vehicle (0.1% DMSO, 0.01% EtOH) as control. After 24 hr, total RNA was harvested and analyzed using quantitative real-time PCR. Data are expressed as mean ± SEM. (F) Expression of MCAD in myotubes lacking RIP140 is reduced by treatment with the XCT790. WT and null myoblasts were differentiated as described. Subsequently, the myotubes were treated with an inverse agonist for ERRα (XCT790; 10 μM) or vehicle (0.1% DMSO) as control. After 48 hr, total RNA was harvested and analyzed using quantitative real-time PCR. (G) Chromatin immunoprecipitation of the FABP3 and MCAD promoters in differentiated WT (RIP140^+/+^) or RIP140 null (−/−) myoblasts with control (IgG) or RIP140 antibody.

A number of nuclear receptors, including PPARδ and ERRα, have also been implicated in the control of mitochondrial activity, and so we investigated their expression as well as that of some of their target genes involved in fatty-acid oxidation in the gastrocnemius and soleus muscles (Tables S4 and S5). FABP3 was one of a number of target genes that was increased in the gastrocnemius in the absence of RIP140 and decreased in the soleus muscle from RIP140 transgenic mice (Figure 4A). There was no alteration in PPARδ expression (Figure 4A), but the levels of PGC-1α decreased by 30%–40% in both muscle types. Importantly, the removal of RIP140 in the gastrocnemius leads to expression levels of FABP3 equivalent to those normally observed in the soleus; conversely overexpression of RIP140 in the soleus in the transgenic mice leads to a decrease in FABP3 expression with levels similar to those found in the gastrocnemius. A number of ERRα target genes were regulated similarly including medium chain acylcoenzyme A dehydrogenase (MCAD), although the expression of ERRα is altered by changes in RIP140 expression (Figure 4B, Table S5). The pattern of opposing transcriptional changes between RIP140 null and transgenic mice provides strong evidence that the differentially expressed targets are regulated by RIP140. Furthermore the observation that altering the amount of RIP140 results in a switch in the level of expression of FABP3 and MCAD to that which closely resembles the endogenous expression of these genes in type1/II fibers emphasizes the physiological significance of the regulation by RIP140.

### FABP3 and MCAD Are Direct Targets for RIP140 Repression in Differentiated Myoblasts

To study the intrinsic role of RIP140 in muscle cells we generated conditionally immortal WT and RIP140 null myoblast cell lines that could be induced to differentiate as judged by myotube formation and fusion and the expression of myotube markers such as myogenin and p21 (Andres and Walsh, 1996) (Figures S5 and 4C). In the absence of RIP140 the myotube cells expressed more MyHC I and IIA and a slight reduction of MyHC IIB (Figure 4D), which is consistent with the increased proportion of oxidative fibers in RIP140 null mice. Importantly we observed elevated levels of FABP3 and MCAD in the RIP140 null differentiated myotubes, consistent with our in vivo observations. To explore the potential role of PPARδ and ERRα, respectively, in the differential expression of these genes we used specific receptor ligands. Treatment of WT myotubes with 9-*cis* retinoic acid and the PPARδ agonist GW501516 increased the expression of FABP3 as shown previously (Dressel et al., 2003). Importantly the upregulation of FABP3 was more marked in cells lacking RIP140 (Figure 4E), supporting the hypothesis that RIP140 is recruited to PPARδ/RXR heterodimers in a ligand-dependent manner to repress transcription. To investigate the role of ERRα we used the inverse agonist XCT790 (Willy et al., 2004), which reduced the expression of MCAD in null cells to the levels found in cells expressing RIP140 (Figure 4F), suggesting that enhanced activity of ERRα is responsible for the increased expression of MCAD observed in RIP140 null myotubes.

To determine whether FABP3 or MCAD are direct targets for repression by RIP140 we carried out chromatin immunoprecipitation assays. Although the promoter for the *fabp3* gene has yet to be fully characterized we noted a direct repeat element with strong homology to a PPARE or RAR binding site approximately 900 base pairs upstream of the transcription start site. Primers were designed around this putative PPARE and also around the nuclear receptor response element 1 (NRRE-1), located in the MCAD promoter, which has been previously shown to be important in the ERRα-mediated regulation of this gene (Hainline et al., 1993; Kamei et al., 2003; Sladek et al., 1997). An antibody specific for RIP140 precipitated the FABP3 and MCAD promoter in differentiated WT myoblasts but not RIP140 null cells (Figure 4G). Thus RIP140 is recruited to FABP3 and MCAD promoters in differentiated myoblasts.

## Discussion

The metabolic activity and fiber-type composition of individual muscle groups are highly responsive to surrounding environmental cues. In this paper, we propose that the corepressor RIP140 may play an important role in regulating programs of gene expression in skeletal muscle relating to metabolic activity and fiber type. Using genetically modified mice we examined the consequences of both gain and loss of RIP140 function and found that the relative levels of RIP140 are a key determinant of these processes. In the absence of the corepressor, muscles exhibited a marked increase in mitochondrial activity accompanied by a corresponding shift in myofibers favoring the more oxidative types. Conversely, increased expression of RIP140 resulted in a decrease in both mitochondrial activity and the number of oxidative myofibers.

Expression profiling identified many metabolic genes, including genes involved in mitochondrial biogenesis, fatty-acid oxidation, and oxidative phosphorylation, that were increased in the absence of RIP140, consistent with the function of RIP140 as a corepressor. Increased metabolic gene expression was supported by whole-animal physiological studies that demonstrated increased fat utilization and energy expenditure in RIP140 null mice relative to WT mice. Interestingly, these changes persist after long-term high-fat feeding with increased flux through these metabolic pathways, suggesting that a normal response to changes in nutrition is maintained in the absence of RIP140. Although the contribution of RIP140 expression in skeletal muscle to whole-animal physiology is still to be determined, gene expression analysis indicates that the repressive effects of RIP140 are maintained in cultured myoblasts, indicating that the corepressor functions as an intrinsic regulator of genes involved in catabolic pathways in muscle.

Consistent with the metabolic changes, the relative proportion of oxidative fibers varies according to the RIP140 expression level. The increase in oxidative capacity found in the edl muscle of RIP140 null mice is accompanied by an increase in the proportion of type IIA and IIX expressing fibers at the expense of IIB fibers. While the changes in MyHC proteins are relatively modest, there is an increase in IIX relative to IIB in the absence of RIP140 together with increased myoglobin and reduced AMPKγ3 expression, markers of oxidative and glycolytic fibers, respectively. Interestingly overexpression of RIP140 in transgenic mice does not result in impaired muscle fiber remodeling in response to voluntary exercise. Several lines of evidence point to a specific regulatory role for RIP140 in the composition and function of myofibers rather than a more extensive role in the developmental regulation of myogenic transcription. First, skeletal muscle from mice lacking RIP140 shows no major differences in the expression of key early and late myogenic markers. Second, the absence of RIP140 in differentiated myoblasts in vitro results in increased levels of expression of MyHC I and reduced expression of MyHC IIB with minimal changes in the myogenic program. Third, the variation in level of RIP140 expression between RIP140 null, heterozygous, WT, and RIP140 transgenic mice results in a pattern of gene expression that parallels the transcriptional program normally found in different types of muscle, which vary in the level of their endogenous RIP140 expression.

PPARδ and the coactivator PGC-1α have been established as important factors that promote mitochondrial activity and fiber-type switching (Leone et al., 2005; Lin et al., 2002; Wang et al., 2004). Both PPARδ and PGC-1α transgenic mice exhibit a switch from reductive to oxidative fibers, and muscle-specific ablation of PGC-1α leads to reduced expression of MyHC I and IIA in oxidative fibers and impaired exercise capacity (Handschin et al., 2007). Expression of PGC-1β in transgenic mice leads to mitochondrial biogenesis, a decrease in fiber size, and, in particular, induction of fast-twitch oxidative type IIX fibers (Arany et al., 2007) while PGC-1β null mice exhibit a reduction in mitochondrial volume in soleus muscle (Lelliott et al., 2006). Thus, the PGC1 coactivators and the RIP140 corepressor seem to promote opposing physiological functions with increased mitochondrial activity resulting from exogenous coactivator expression or reduced RIP140 expression. On the other hand, reduced mitochondrial activity is observed in the soleus of mice devoid of PGC-1 coactivators or in RIP140 transgenic mice. Thus it appears that PGC-1 coactivators and the RIP140 corepressor serve mutually antagonistic functions in the regulation of mitochondrial activity and, to a lesser extent, fiber-type composition.

We propose that the ability of RIP140 to suppress metabolic activity in skeletal muscle is achieved, at least in part, by its recruitment to nuclear receptors that regulate transcription from specific gene clusters. To investigate the role of RIP140 in the regulation of metabolic processes in skeletal muscle we focused on two key genes in fatty-acid oxidation, namely FABP3 and MCAD, both of which are upregulated in skeletal muscle in the absence of RIP140. FABP3 belongs to a multigene family of intracellular proteins involved in fatty-acid transport and utilization (Veerkamp and Maatman, 1995), and MCAD mediates the initial step in fatty-acid beta-oxidation (Hainline et al., 1993). The expression of these genes seems to reflect the capacity of metabolic tissues to oxidize fatty acids (Hainline et al., 1993; Veerkamp and van Moerkerk, 1993). FABP3 is regulated by PPARδ while MCAD is regulated by ERRα (Sladek et al., 1997; Mootha et al., 2004), and so it was possible to address the role of these receptors in mediating transcriptional repression by RIP140. Chromatin immunoprecipitation assays demonstrated that RIP140 was recruited to the promoters for both FABP3 and MCAD, suggesting that they are direct targets for the corepressor. RIP140 bound in the vicinity of a putative response element for PPARs in the FABP3 promoter that is conserved in both mice and rats and to a nuclear receptor response element located in the MCAD promoter (Hainline et al., 1993; Kamei et al., 2003; Sladek et al., 1997). We were able to confirm that the ability of PPARδ to stimulate FABP3 expression was maintained in differentiated myoblasts, but importantly the increase was greater in cells devoid of RIP140. Interestingly treatment with 9-*cis* RA, a ligand for RXR, also potentiated FABP3 expression to a greater extent in the absence of RIP140. These observations, together with the chromatin immunoprecipitation experiments, suggest that RIP140 recruitment to PPARδ-RXR heterodimers is responsible for repressing FABP3 expression in skeletal muscle. The ability of ERRα to activate transcription from target genes is dependent on the recruitment of PGC-1α or PGC-1β (Kamei et al., 2003; Mootha et al., 2004). That ERRα is a direct target for RIP140 is supported by the observation that myotubes devoid of the corepressor express increased levels of MCAD and that this increase was blocked by a specific inverse agonist for this receptor. The depletion of RIP140 is, however, accompanied by an increase in ERRα expression, which may also contribute to the upregulation of MCAD. Thus it appears that the regulation of metabolic gene networks by PPARδ and ERRα may be determined by the relative levels or activity of PGC-1 coactivators and the RIP140 corepressor.

Previous studies have shown that RIP140 represses metabolic gene networks in white adipose tissue where lack of the corepressor results in increased expression of many genes, including UCP1, a protein normally only expressed in brown adipose tissue (Leonardsson et al., 2004; Powelka et al., 2006). The induction of UCP1 and the conversion of white adipocytes to a brown fat phenotype has also been achieved by the ectopic expression of PGC-1α (Puigserver et al., 1998). It appears therefore that RIP140 and PGC1α have mutually antagonizing roles in the regulation of metabolism in both muscle and fat and potentially other tissues where the cofactors are coexpressed. It is conceivable that such antagonism may be regulated by alterations in the relative expression of the cofactors, posttranslational modifications (Christian et al., 2006; Gerhart-Hines et al., 2007), or their subcellular localization (Lerin et al., 2006). Therefore it is conceivable that RIP140 may function in combination with specific isoforms of PGC-1 and with nuclear receptors such as PPARδ and ERRα in signaling pathways that promote fiber-type transitions.

Several animal models have shown that muscle fiber-type transformation is associated with a shift in the metabolic parameters to a more insulin-responsive phenotype and protects against the development of diet-induced glucose intolerance (Ryder et al., 2003; Wang et al., 2004). In keeping with this we have shown that mice lacking RIP140 are protected from the insulin resistance associated with high-fat feeding or aging and this is due to an increase in insulin sensitivity (Powelka et al., 2006). In conclusion, it is clear from this study that the precise level of expression of RIP140, as shown by the progressive changes observed in null, heterozygous, WT, and overexpressing transgenic animals, is an important factor in muscle function and physiology. It is conceivable that modulation of RIP140 signaling in muscle may be an important target in the treatment of insulin resistance and type 2 diabetes.

## Experimental Procedures

### Animals

The generation of RIP140 null mice has previously been described (White et al., 2000). Mice used in this study were backcrossed eight generations to C57BL/6J background, except those used for the microarray profiling that were on a mixed or a high-fat diet (45% kcal) (Research Diets, NJ), as indicated.

RIP140 transgenic mice were generated using the human RIP140 coding sequence fused to a β-globin polyA sequence and placed downstream of a CMV enhancer and chicken β-actin promoter. From six independent transgenic mouse lines, two were studied in detail and data from line 51 are presented here.

RIP140 null mice were crossed with H-2K^b^ –tsA58 transgenic mice expressing an inducible temperature-sensitive version of SV40 T antigen (Jat et al., 1991), and second generation WT or RIP140 null offspring were used to generate myoblast cultures.

In voluntary exercise studies male mice aged 3 weeks were housed in groups of three animals and allowed constant access to exercise wheels (Bio-Serv) for a total of 6 weeks. Control groups were housed in the absence of wheels for the duration of the experiment.

Oxygen consumption and carbon dioxide production were simultaneously determined in an Oxymax metabolic chamber system by indirect calorimetry (Columbus Instruments) (n = 5–6 per group).

All animal studies were carried out according to UK Home Office guidelines.

### Myoblast Cell Cultures

Conditionally immortal myogenic clonal cell lines were derived from the edl as previously described (Morgan et al., 1994). The effects of nuclear receptor ligands on gene expression in differentiated myoblasts (4 days) were investigated by the addition of agonists for PPARδ (GW501516, 1 μM), RXR (9-*cis* retinoic acid [100 nM Sigma]), ERRα (XCT790, 10 μM), or vehicle (0.1% DMSO and 0.01% EtOH) as control for 24 hr.

### Histochemistry

Histochemical analyses were carried out for SDH and metachromatic ATPase as previously described (Ogilvie and Feeback, 1990). Fiber types in soleus, extensor digitorum longus, and gastrocnemius were characterized by MyHC immunostaining using primary monoclonal mouse antibodies against type I (BAF-8), IIA (SC-71), IIB (BFF-3), IIX (6H1, from Joseph Hoh), or all type II (Sigma anti-fast) myosin isoforms. Detailed methods are described in the Supplemental Data.

### Expression Analysis

The expression of RIP140 and L-19 was determined with specific primers and Taqman probes, while the expression of other genes was determined using SYBR green reagent and gene-specific primers. Expression levels were normalized against the expression of the ribosomal coding gene L-19. Primer sequences can be obtained on request.

### Transmission Electron Microscopy

Edl samples were fixed for 24 hr in 3% v/v glutaraldehyde in 0.1 M cacodylate buffer (pH 7.3), post-fixed in osmium tetroxide, and embedded in Araldite. Ultra-thin sections were examined using a Philips CM10 TEM (FEI, Cambridge).

### Affymetrix Microarray Hybridization and Data Analysis

Expression profiling was performed using Murine 430 2.0 chips. Hybridization and scanning were performed by the CSC/IC Microarray Centre (Imperial College London). Data were analyzed with d-CHIP software (Li and Wong, 2001). p values were generated in d-CHIP by a two-tailed unpaired Student's t test to use as a ranking and filtering device. The microarray data are available at http://www.ebi.ac.uk/arrayexpress/ under accession number E-BAIR-13.

### Chromatin Immunoprecipitation Assay

Differentiated myoblasts (day 5) were crosslinked and immunoprecipitated as described previously (Christian et al., 2005) using anti-RIP140 (a gift from Dr D. Chen). Primer sequences are available on request.

## Supplemental Data

Document S1. Supplemental Experimental Procedures, Five Figures, and Six Tables

## References

[bib1] Andres V., Walsh K. (1996). Myogenin expression, cell cycle withdrawal, and phenotypic differentiation are temporally separable events that precede cell fusion upon myogenesis. J. Cell Biol..

[bib2] Arany Z., Lebrasseur N., Morris C., Smith E., Yang W., Ma Y., Chin S., Spiegelman B.M. (2007). The transcriptional coactivator PGC-1beta drives the formation of oxidative type IIX fibers in skeletal muscle. Cell Metab..

[bib3] Baldwin K.M., Haddad F. (2001). Effects of different activity and inactivity paradigms on myosin heavy chain gene expression in striated muscle. J. Appl. Physiol..

[bib4] Bassel-Duby R., Olson E.N. (2006). Signaling pathways in skeletal muscle remodeling. Annu. Rev. Biochem..

[bib5] Cavailles V., Dauvois S., L'Horset F., Lopez G., Hoare S., Kushner P.J., Parker M.G. (1995). Nuclear factor RIP140 modulates transcriptional activation by the estrogen receptor. EMBO J..

[bib6] Chin E.R., Olson E.N., Richardson J.A., Yang Q., Humphries C., Shelton J.M., Wu H., Zhu W., Bassel-Duby R., Williams R.S. (1998). A calcineurin-dependent transcriptional pathway controls skeletal muscle fiber type. Genes Dev..

[bib7] Christian M., Kiskinis E., Debevec D., Leonardsson G., White R., Parker M.G. (2005). RIP140-targeted repression of gene expression in adipocytes. Mol. Cell. Biol..

[bib8] Christian M., White R., Parker M.G. (2006). Metabolic regulation by the nuclear receptor corepressor RIP140. Trends in endocrinology and metabolism. Trends Endocrinol. Metab..

[bib9] Dressel U., Allen T.L., Pippal J.B., Rohde P.R., Lau P., Muscat G.E. (2003). The peroxisome proliferator-activated receptor beta/delta agonist, GW501516, regulates the expression of genes involved in lipid catabolism and energy uncoupling in skeletal muscle cells. Mol. Endocrinol..

[bib10] Gerhart-Hines Z., Rodgers J.T., Bare O., Lerin C., Kim S.H., Mostoslavsky R., Alt F.W., Wu Z., Puigserver P. (2007). Metabolic control of muscle mitochondrial function and fatty acid oxidation through SIRT1/PGC-1alpha. EMBO J..

[bib11] Hainline B.E., Kahlenbeck D.J., Grant J., Strauss A.W. (1993). Tissue specific and developmental expression of rat long-and medium-chain acyl-CoA dehydrogenases. Biochim. Biophys. Acta.

[bib12] Handschin C., Kobayashi Y.M., Chin S., Seale P., Campbell K.P., Spiegelman B.M. (2007). PGC-1alpha regulates the neuromuscular junction program and ameliorates Duchenne muscular dystrophy. Genes Dev..

[bib13] Huss J.M., Torra I.P., Staels B., Giguere V., Kelly D.P. (2004). Estrogen-related receptor alpha directs peroxisome proliferator-activated receptor alpha signaling in the transcriptional control of energy metabolism in cardiac and skeletal muscle. Mol. Cell. Biol..

[bib14] Jat P.S., Noble M.D., Ataliotis P., Tanaka Y., Yannoutsos N., Larsen L., Kioussis D. (1991). Direct derivation of conditionally immortal cell lines from an H-2Kb-tsA58 transgenic mouse. Proc. Natl. Acad. Sci. USA.

[bib15] Kamei Y., Ohizumi H., Fujitani Y., Nemoto T., Tanaka T., Takahashi N., Kawada T., Miyoshi M., Ezaki O., Kakizuka A. (2003). PPARgamma coactivator 1beta/ERR ligand 1 is an ERR protein ligand, whose expression induces a high-energy expenditure and antagonizes obesity. Proc. Natl. Acad. Sci. USA.

[bib16] L'Horset F., Dauvois S., Heery D.M., Cavailles V., Parker M.G. (1996). RIP-140 interacts with multiple nuclear receptors by means of two distinct sites. Mol. Cell. Biol..

[bib17] Larsson L., Edstrom L., Lindegren B., Gorza L., Schiaffino S. (1991). MHC composition and enzyme-histochemical and physiological properties of a novel fast-twitch motor unit type. Am. J. Physiol..

[bib18] Lelliott C.J., Medina-Gomez G., Petrovic N., Kis A., Feldmann H.M., Bjursell M., Parker N., Curtis K., Campbell M., Hu P. (2006). Ablation of PGC-1beta results in defective mitochondrial activity, thermogenesis, hepatic function, and cardiac performance. PLoS Biol..

[bib19] Leonardsson G., Steel J.H., Christian M., Pocock V., Milligan S., Bell J., So P.W., Medina-Gomez G., Vidal-Puig A., White R., Parker M.G. (2004). Nuclear receptor corepressor RIP140 regulates fat accumulation. Proc. Natl. Acad. Sci. USA.

[bib20] Leone T.C., Lehman J.J., Finck B.N., Schaeffer P.J., Wende A.R., Boudina S., Courtois M., Wozniak D.F., Sambandam N., Bernal-Mizrachi C. (2005). PGC-1alpha deficiency causes multi-system energy metabolic derangements: muscle dysfunction, abnormal weight control and hepatic steatosis. PLoS Biol..

[bib21] Lerin C., Rodgers J.T., Kalume D.E., Kim S.H., Pandey A., Puigserver P. (2006). GCN5 acetyltransferase complex controls glucose metabolism through transcriptional repression of PGC-1alpha. Cell Metab..

[bib22] Li C., Wong W.H. (2001). Model-based analysis of oligonucleotide arrays: expression index computation and outlier detection. Proc. Natl. Acad. Sci. USA.

[bib23] Lin J., Wu H., Tarr P.T., Zhang C.Y., Wu Z., Boss O., Michael L.F., Puigserver P., Isotani E., Olson E.N. (2002). Transcriptional co-activator PGC-1 alpha drives the formation of slow-twitch muscle fibres. Nature.

[bib24] Lucas C.A., Kang L.H., Hoh J.F. (2000). Monospecific antibodies against the three mammalian fast limb myosin heavy chains. Biochem. Biophys. Res. Commun..

[bib25] McCullagh K.J., Calabria E., Pallafacchina G., Ciciliot S., Serrano A.L., Argentini C., Kalhovde J.M., Lomo T., Schiaffino S. (2004). NFAT is a nerve activity sensor in skeletal muscle and controls activity-dependent myosin switching. Proc. Natl. Acad. Sci. USA.

[bib26] Mootha V.K., Handschin C., Arlow D., Xie X., St Pierre J., Sihag S., Yang W., Altshuler D., Puigserver P., Patterson N. (2004). Erralpha and Gabpa/b specify PGC-1alpha-dependent oxidative phosphorylation gene expression that is altered in diabetic muscle. Proc. Natl. Acad. Sci. USA.

[bib27] Morgan J.E., Beauchamp J.R., Pagel C.N., Peckham M., Ataliotis P., Jat P.S., Noble M.D., Farmer K., Partridge T.A. (1994). Myogenic cell lines derived from transgenic mice carrying a thermolabile T antigen: a model system for the derivation of tissue-specific and mutation-specific cell lines. Dev. Biol..

[bib28] Ogilvie R.W., Feeback D.L. (1990). A metachromatic dye-ATPase method for the simultaneous identification of skeletal muscle fiber types I, IIA, IIB and IIC. Stain Technol..

[bib29] Olson E.N., Williams R.S. (2000). Remodeling muscles with calcineurin. Bioessays.

[bib30] Powelka A.M., Seth A., Virbasius J.V., Kiskinis E., Nicoloro S.M., Guilherme A., Tang X., Straubhaar J., Cherniack A.D., Parker M.G., Czech M.P. (2006). Suppression of oxidative metabolism and mitochondrial biogenesis by the transcriptional corepressor RIP140 in mouse adipocytes. J. Clin. Invest..

[bib31] Puigserver P., Wu Z., Park C.W., Graves R., Wright M., Spiegelman B.M. (1998). A cold-inducible coactivator of nuclear receptors linked to adaptive thermogenesis. Cell.

[bib32] Ryder J.W., Bassel-Duby R., Olson E.N., Zierath J.R. (2003). Skeletal muscle reprogramming by activation of calcineurin improves insulin action on metabolic pathways. J. Biol. Chem..

[bib33] Schiaffino S., Reggiani C. (1996). Molecular diversity of myofibrillar proteins: gene regulation and functional significance. Physiol. Rev..

[bib34] Sladek R., Bader J.A., Giguere V. (1997). The orphan nuclear receptor estrogen-related receptor alpha is a transcriptional regulator of the human medium-chain acyl coenzyme A dehydrogenase gene. Mol. Cell. Biol..

[bib35] van Breda E., Keizer H.A., Vork M.M., Surtel D.A., de Jong Y.F., van der Vusse G.J., Glatz J.F. (1992). Modulation of fatty-acid-binding protein content of rat heart and skeletal muscle by endurance training and testosterone treatment. Pflugers Arch..

[bib36] Veerkamp J.H., van Moerkerk H.T. (1993). Fatty acid-binding protein and its relation to fatty acid oxidation. Mol. Cell. Biochem..

[bib37] Veerkamp J.H., Maatman R.G. (1995). Cytoplasmic fatty acid-binding proteins: their structure and genes. Prog. Lipid Res..

[bib38] Wang Y.X., Zhang C.L., Yu R.T., Cho H.K., Nelson M.C., Bayuga-Ocampo C.R., Ham J., Kang H., Evans R.M. (2004). Regulation of muscle fiber type and running endurance by PPARdelta. PLoS Biol..

[bib39] White R., Leonardsson G., Rosewell I., Ann Jacobs M., Milligan S., Parker M. (2000). The nuclear receptor co-repressor nrip1 (RIP140) is essential for female fertility. Nat. Med..

[bib40] Willy P.J., Murray I.R., Qian J., Busch B.B., Stevens W.C., Martin R., Mohan R., Zhou S., Ordentlich P., Wei P. (2004). Regulation of PPARgamma coactivator 1alpha (PGC-1alpha) signaling by an estrogen-related receptor alpha (ERRalpha) ligand. Proc. Natl. Acad. Sci. USA.

[bib41] Wu H., Gallardo T., Olson E.N., Williams R.S., Shohet R.V. (2003). Transcriptional analysis of mouse skeletal myofiber diversity and adaptation to endurance exercise. J. Muscle Res. Cell Motil..

[bib42] Wu Z., Puigserver P., Andersson U., Zhang C., Adelmant G., Mootha V., Troy A., Cinti S., Lowell B., Scarpulla R.C., Spiegelman B.M. (1999). Mechanisms controlling mitochondrial biogenesis and respiration through the thermogenic coactivator PGC-1. Cell.

