# Supplementary material for: The Transcriptional Corepressor RIP140 Regulates Oxidative Metabolism in Skeletal Muscle
Source: Cell Metab. 2007 Sep 5;6(3):236–45. doi: 10.1016/j.cmet.2007.08.004 (PMC2680991; doi:10.1016/j.cmet.2007.08.004)
Supplement: Document S1. Supplemental Experimental Procedures, Five Figures, and Six Tables [file mmc1.pdf]

Cell Metabolism, Volume *6*

Supplemental Data

The Transcriptional Corepressor RIP140 Regulates Oxidative Metabolism in Skeletal Muscle

Asha Seth, Jennifer H. Steel, Donna Nichol, Victoria Pocock, Mande K. Kumaran, Asmaa Fritah, Margaret Mobberley, Timothy A. Ryder, Anthea Rowlerson, James Scott, Matti Poutanen, Roger White, and Malcolm Parker

Supplemental Experimental Procedures

DNA extraction and determination of mitochondrial copy

DNA was extracted from edl of wild type and null mice using a DNAeasy kit (Qiagen). Real-time PCR with SYBR green reagent and specific primers was used to monitor levels of the mitochondrial cytochrome C oxidase subunit II gene and normalised to levels of the nucleus-encoded UCP1 promoter.

Histochemical staining for succinate dehydrogenase.

Muscle samples were excised and immediately frozen in liquid N_2. Frozen sections (10 µm) of unfixed muscle were cut in a cryostat and mounted onto poly-L-lysine coated glass slides. Sections were allowed to dry at room temperature for 1 hour then stained for succinate dehydrogenase for 5 min. Sections were mounted in glycerol gelatin (Sigma).

Characterisation of muscle fibre types.

Fibre types in soleus, extensor digitorum longus and gastrocnemius were characterised using a combination of metachromatic ATPase and myosin heavy chain immunostaining. RIP140-null and wild-type muscles were embedded in the same block for histological analysis to control for any variations in staining and serial frozen sections (10 µm) were cut. The pH stability of myosin ATPase was used to determine the proportions of each fibre type as previously described (Ogilvie and Feeback, 1990). Sections were also immunostained using primary monoclonal mouse antibodies against slow type I (BAF-8), type IIA (SC-71), type IIB (BFF-3) or all type II (Sigma anti-fast) myosin isoforms and indirect immunoperoxidase. The type IIX antibody was a generous gift of Joseph Hoh. Negative control slides with omission of the primary antibody were included in each immunostaining procedure. The separation of myosin isoforms was carried out essentially as described in (REF) using 0.5ug total muscle protein on 8% polyacrlamide gels containing 30% glycerol run at a constant 70V for 24 hours. Myosin proteins were detected by silver staining using the Proteosilver Plus Silver Stain kit (Sigma, Poole UK).

Affymetrix microarray hybridisation and data analysis.
Total RNA was isolated from the gastrocnemius of wild type or null animals fed either control diet (10% kcal) or high fat diet (45% kcal) for 3 months (*n*=5,6). Affymetrix array hybridization and scanning were performed by the CSC/IC Microarray Centre, Imperial College London, Hammersmith Campus, using murine 430 2.0 chips. Array data were analyzed with d-CHIP software (Li and Wong, 2001). P-values were generated in d-CHIP by a 2 tailed unpaired student's t-test to use as a ranking and filtering device. The p-values were not adjusted for multiple testing.

Cell culture of H2K cells. Conditionally immortal myogenic clonal cell lines were derived from the edl of H2K x RIP140+/+ and -/- mice as previously described (Morgan et al., 1994). Cell proliferation and differentiation of these cells are temperature and interferon (IFN)-γ-dependent. Myoblasts were maintained in the presence of heat-inactivated fetal calf serum (20%), chick embryo extract (2%), gelatin (0.01%) and IFN-γ at 33˚C. In order to differentiate into myotubes cells were plated densely, switched to media without IFN-γ and low serum (5% horse serum) and the temperature was raised to 37˚C. This resulted in sarcomere formation within 3-4 days.

Ligand Treatment.
Wild type and null cells were differentiated into myotubes for 4 days and the medium was changed to 2% horse serum supplemented with the agonists for PPARδ (GW501516, 1μM), RXR (9-cis retinoic acid (100 nM Sigma)) or the vehicle (0.1% DMSO and 0.01% EtOH) as control. Cells were harvested after 24 hrs. With ERRα ligand cells were differentiated for 3 days and the medium was changed to 2% horse serum supplemented with XCT790 (10μM, a gift from Tim Willson and William J. Zuercher, GlaxoSmithKline) or the vehicle (0.1% DMSO) as control. Cells were harvested after 48 hrs.

Chromatin immunoprecipitation assay
Fully differentiated cells (day 5) were incubated in Dimethyl adipimidate 2 (DMA) in media (final concentration 10mM) for 30 mins at room temperature to cross-link protein-protein interactions. The cells were put in 1% formaldehyde in PBS for 15 mins at 37 ºC to cross link protein and DNA. Cross-linked cells were lysed, sonicated and immunoprecipitated with protein A/G PLUS-agarose (Santa Cruz, SC-2003) according to the manufacturers instructions using rabbit-polyclonal anti-mouse RIP140 (a gift from Dr D. Chen) or control normal rabbit IgG (Santa Cruz SC-2027). DNA fragments were purified with a QIAquick PCR purification kit (QIAGEN) and used as templates for PCR. Primer sequences are available on request.

Seth: Supplementary Fig 1

A. SDH staining in wt, heterozygous and null gastrocnemius

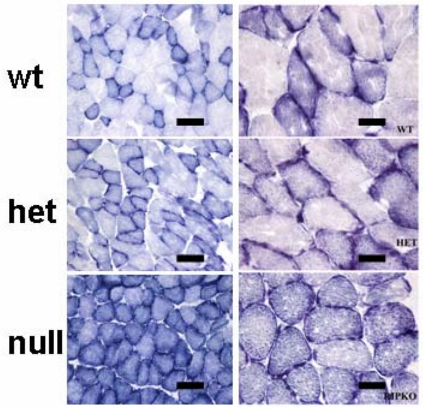

Figure S1.
A. SDH staining in wt, het and null gastrocnemius sections
Histochemical staining for SDH in muscle sections at lower and higher magnification (scale bars=100µm at low magnification and 40µm at high magnification)

Seth: Supplementary Fig 2

A. Semi-quantitative evaluation of number of mitochondria in wt, heterozygous and null edl

	wt	het	ko
1	1.65	2.05	3.05
2	1.10	1.65	2.75
3	1.40	2.25	2.95

B. Cytochrome C oxidase subunit II DNA (MTCO2)

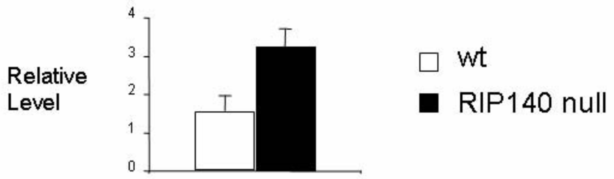

Figure S2.

A. Semi-quantitative evaluation of mitochondria in wt, heterozygous and null muscle

Scanning Electron Microscopy was carried out on muscle sections taken from the EDL of wt, het and null mice. A scoring system was devised to semi-quantify the number of mitochondria per skeletal muscle fibre with 1 being the least (pairs of mitochondria uniformly distributed near the z line) to 4 (skeletal muscle fibres with long strings of enlarged mitochondria found throughout the field of view). The scores are an average of 20 different skeletal muscle fibres scored blind by 4 individuals.

B. Quantification of mitochondrial copy number

The EDL was dissected from adult, age-matched wt and RIP140 null mice and DNA was extracted using a DNAeasy kit (QIAGEN). Real-time PCR with SYBR green reagent and specific primers was used to monitor levels of the mitochondrial cytochrome c oxidase subunit II gene. Values are shown as normalised to levels of the nucleus-encoded UCP1 promoter (n=3).

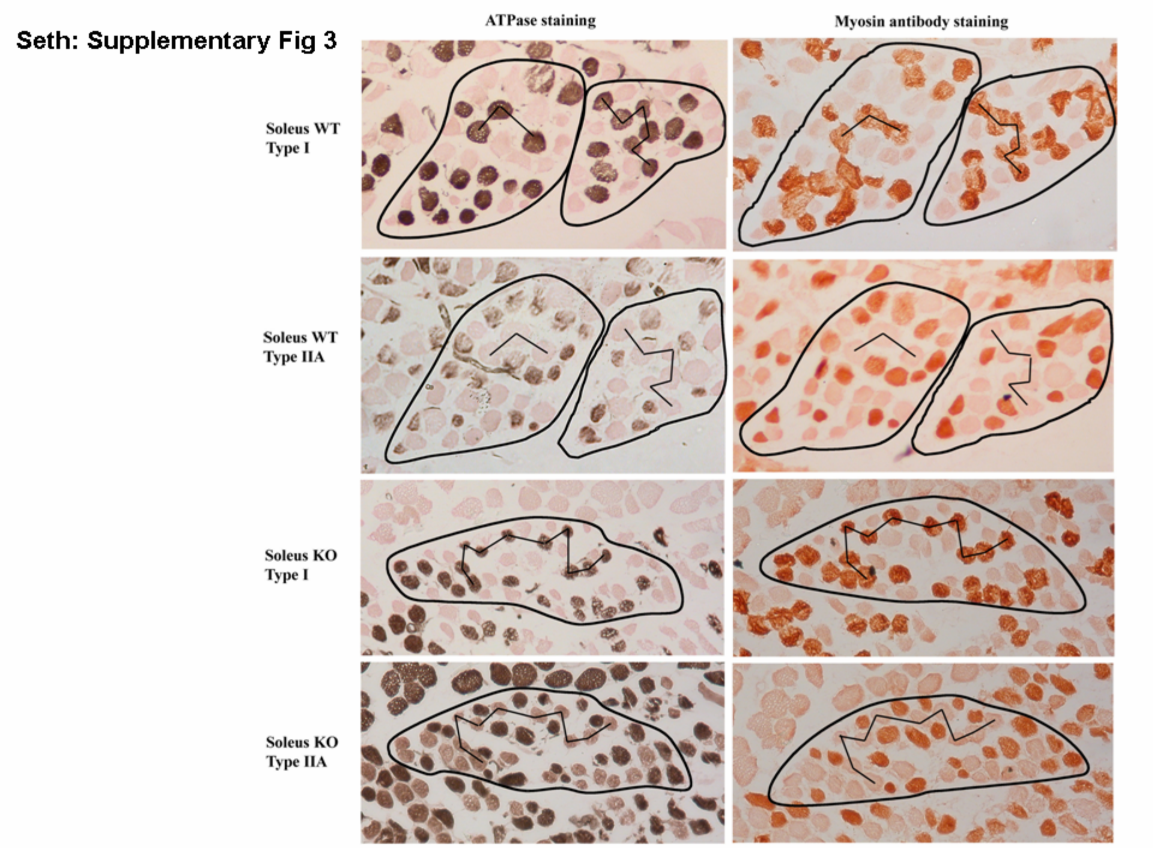

Figure S3. Analysis of muscle fibre types by metachromatic ATPase and myosin heavy chain immunostaining.
Serial sections of soleus muscle from wild type and RIP140 null mice were examined by metachromatic ATPase and myosin heavy chain immunostaining to determine the numbers of fibres corresponding to different types of fibres. The areas marked are for myofibre identification purposes only. Immunostaining for type I and type IIA fibres is shown with a line drawn through the type I fibres to facilitate the distinction with type IIA fibres.

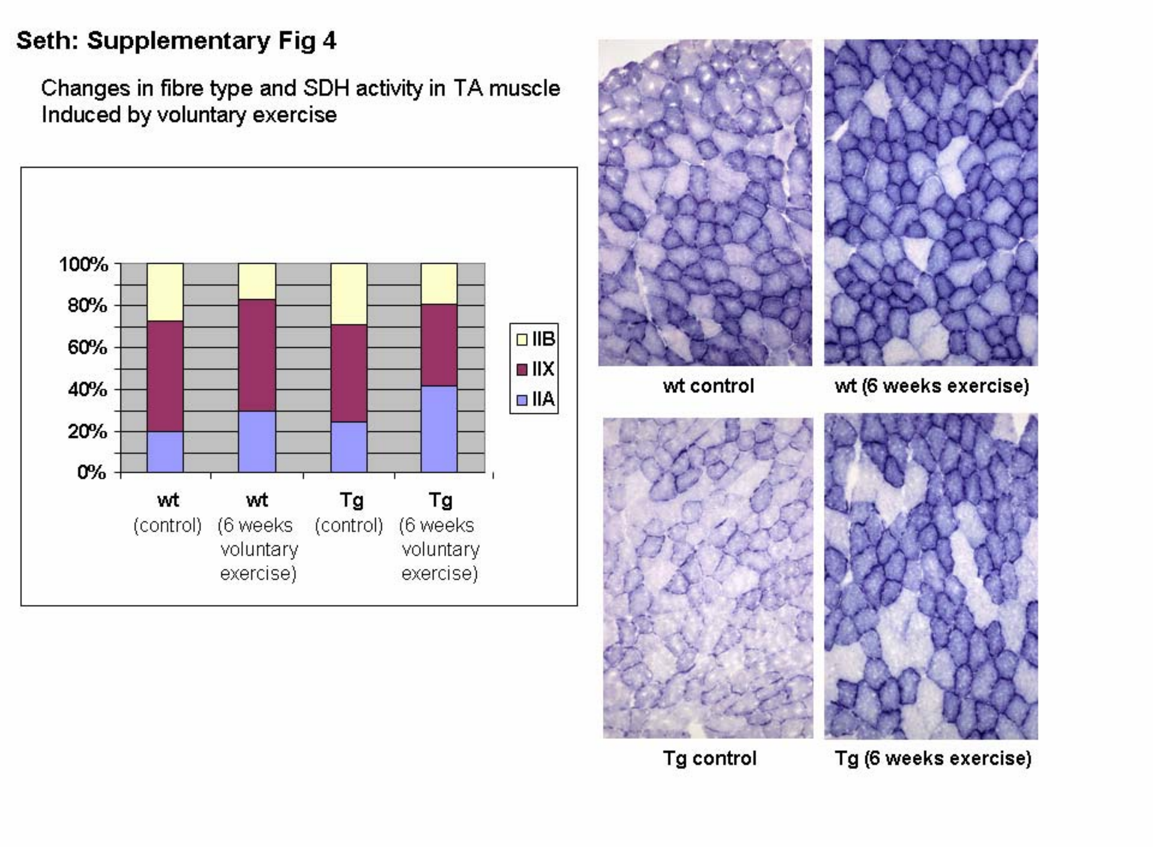

Figure S4. Analysis of fibre type composition and SDH staining in TA muscle after exercise. TA muscle from wild type and RIP140Tg mice either untrained or allowed free access to voluntary exercise wheels for 6 weeks was exanibed for MyHC expression and SDH activity. The histograms show the proportional change in fibre type as determined by detection of IIA, IIX and IIB stained fibres using specific antibodies

Light microscopy of proliferating wild type and RIP140 null myoblasts and differentiated myotubes.

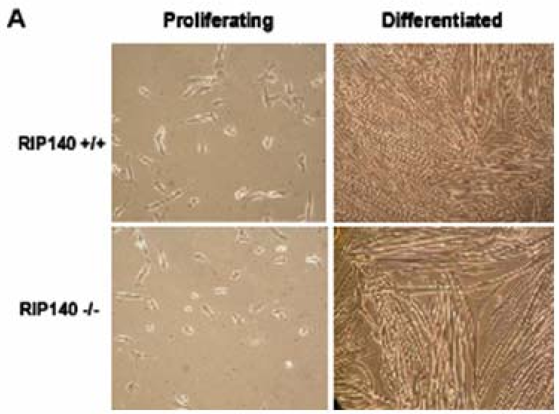

Figure S5.
Light microscopy of proliferating wild type and RIP140 null myoblasts and differentiated myotubes. Proliferating myoblasts were grown at low density in 20% fetal bovine serum and induced to differentiate by culturing in 5% horse serum for five days.

Table S1. Relative proportions of muscle fibre types in the soleus and extensor digitorum longus (EDL) muscles of wild-type and RIP140-null mice

Muscle	Fibre Type	WT	RIP140-null
EDL	IIA	11.3 ± 2.8 (4)	19.9 ± 4.5 (4
	IIX	23.8 ± 4.4 (4)	32.4 ± 4.0 (4)
	IIB	64.9 ± 5.6 (4)	46.5 ± 2.0 (4)
Soleus	I	36.6 ± 2.2 (3)	46.8 ± 4.7 (3)
	IIA	57.7 ± 1.5 (3)	53.2 ± 4.7 (3)
	IIX	5.7 ± 0.7 (3)	0 (3)

Serial sections were analysed by metachromatic ATPase and myosin heavy chain immunostaining as shown in Supplementary Fig 1 to quantitate type I, IIA and IIB fibres while the proportion of type IIX fibres was deduced from the difference between total type II and type IIA plus IIB fibres and confirmed using the specific IIX antibody 6H1. Values are the means ± SEM from 3 or 4 independent analyses as indicated. Changes in the proportion of type I fibres in the soleus and type II fibres in the EDL were significant at $P < 0.05$.

Table S2. Muscle-specific genes significantly altered by absence of RIP140

probe set	gene	fold change	P value
1416023_at	fatty acid binding protein 3, muscle and heart	2.14	0
1452465_at	myosin, heavy polypeptide 1, skeletal muscle, adult	1.93	0.001118
1450511_at	muscle, skeletal, receptor tyrosine kinase	1.58	0.003179
1422529_s_at	calsequestrin 2	1.56	0.030164
1420653_at	transforming growth factor, beta 1	1.54	0.013539
1435463_s_at	RIKEN cDNA 9930104H07 gene	1.46	0.03076
1448962_at	myosin heavy chain 11, smooth muscle	1.42	0.005182
1450196_s_at	glycogen synthase 3, brain	1.4	0.004291
1448327_at	actinin alpha 2	1.37	0.00073
1452670_at	myosin, light polypeptide 9, regulatory	1.34	0.00657
1419150_at	myogenic factor 6	1.33	0.008215
1422544_at	myosin X	1.33	0.054081
1418420_at	myogenic differentiation 1	1.32	0.023761
1423505_at	transgelin	1.32	0.023365
1449551_at	myosin IC	1.25	0.052273
1415927_at	actin, alpha, cardiac	1.24	0.011901
1426750_at	filamin, beta	1.17	0.05491
1449577_x_at	tropomyosin 2, beta	1.12	0.031624
1416780_at	phosphofructokinase, muscle	-1.09	0.05325
1448602_at	muscle glycogen phosphorylase	-1.11	0.02113
1418677_at	actinin alpha 3	-1.13	0.008335
1457435_x_at	myomesin 2	-1.14	0.005234
1417626_at	upregulated during skeletal muscle growth 4	-1.14	0.000877
1442481_at	dual specificity phosphatase 4	-1.16	0.029378
1418066_at	cofilin 2, muscle	-1.17	0.046566
1434996_at	solute carrier family 25 (mitochondrial carrier; Graves disease autoantigen), member 16	-1.2	0.04887
1427943_at	acylphosphatase 2, muscle type	-1.21	0.001441
1420368_at	density-regulated protein	-1.21	0.003869
1422598_at	calsequestrin 1	-1.23	0.000213
1423058_at	capping protein (actin filament) muscle Z-line, alpha 2	-1.25	0.019776
1424168_a_at	capping protein (actin filament) muscle Z-line, beta	-1.26	0.000223
1421028_a_at	myocyte enhancer factor 2C	-1.26	0.037294
1449178_at	PDZ and LIM domain 3	-1.26	0.006366
1416521_at	selenoprotein W, muscle 1	-1.31	0.00197
1440940_at	calcium channel, voltage-dependent, beta 1 subunit	-1.32	0.00277
1456623_at	tropomyosin 1, alpha	-1.37	0.013661
1444083_at	RIKEN cDNA 2310036G12 gene	-1.43	0.001117
1417756_a_at	lymphocyte specific 1	-1.45	0.000921
1435567_at	phosphorylase kinase alpha 1	-1.45	0.000066
1436909_at	RIKEN cDNA B430110G05 gene	-1.5	0.008069
1419487_at	myosin binding protein H	-1.55	0.000399
1457347_at	ryanodine receptor 1, skeletal muscle	-1.75	0.011774

Affymetrix microarray analysis of mRNA levels in wt vs null gastrocnemius muscle in adult mice fed a normal chow diet (*n*=6).

Table S3. Metabolic genes significantly altered by absence of RIP140
A.

Expression of OXPHOS pathway genes

		Fold Change	p-value
1451084_at	electron transferring flavoprotein, dehydrogenase	1.6	0.000027
1448222_x_at	cytochrome c oxidase, subunit VIIIa	1.49	0.00005
1423972_at	electron transferring flavoprotein, alpha polypeptide	1.4	0.000018
1417417_a_at	cytochrome c oxidase, subunit VI a, polypeptide 1	1.34	0.026276
1428631_a_at	RIKubiquinol cytochrome c reductase core protein 2	1.23	0.00066
1426688_at	succinate dehydrogenase complex, subunit A, flavoprotein (Fp)	1.22	0.000935
1418709_at	cytochrome c oxidase, subunit VIIa 1	1.22	0.00024
1416971_at	cytochrome c oxidase, subunit VIIa 2	1.2	0.000138
1416663_at	NADH dehydrogenase (ubiquinone) 1 alpha subcomplex, 9	1.19	0.000895
1451096_at	NADH dehydrogenase (ubiquinone) Fe-S protein 2	1.19	0.003318
1428322_a_at	NADH dehydrogenase (ubiquinone) 1 beta subcomplex, 10	1.18	0.003015
1415967_at	NADH dehydrogenase (ubiquinone) flavoprotein 1	1.18	0.002801
1423692_at	NADH dehydrogenase (ubiquinone) 1 alpha subcomplex, 8	1.17	0.000806
1423711_at	NADH dehydrogenase (ubiquinone) 1 alpha subcomplex, assembly factor 1	1.17	0.019141
1439267_x_at	cytochrome c oxidase, subunit Va	1.17	0.003343
1416604_at	cytochrome c-1	1.16	0.001694
1423907_a_at	NADH dehydrogenase (ubiquinone) Fe-S protein 8	1.15	0.001343
1452692_a_at	NADH dehydrogenase (ubiquinone) flavoprotein 2	1.15	0.002039
1435112_a_at	ATP synthase, H+ transporting, mitochondrial F0 complex, subunit d	1.15	0.009771
1428159_s_at	NADH dehydrogenase (ubiquinone) 1, alpha/beta subcomplex, 1	1.14	0.007804
1425143_a_at	NADH dehydrogenase (ubiquinone) Fe-S protein 1	1.14	0.008381
1428235_at	succinate dehydrogenase complex, subunit D, integral membrane protein	1.14	0.008051
1428181_at	electron transferring flavoprotein, beta polypeptide	1.13	0.031895
1426689_s_at	succinate dehydrogenase complex, subunit A, flavoprotein (Fp)	1.12	0.010738
1448630_a_at	succinate dehydrogenase complex, subunit C, integral membrane protein	1.12	0.003165
1448959_at	NADH dehydrogenase (ubiquinone) Fe-S protein 4	1.1	0.028891
1424313_a_at	NADH dehydrogenase (ubiquinone) Fe-S protein 7	1.1	0.034024
1416269_at	ATP synthase, H+ transporting, mitochondrial F0 complex, subunit f, isoform 2	1.1	0.005106
1428782_a_at	ubiquinol-cytochrome c reductase core protein 1	1.09	0.197333
1417286_at	NADH dehydrogenase (ubiquinone) 1 alpha subcomplex, 5	1.08	0.083829
1416829_at	ATP synthase, H+ transporting mitochondrial F1 complex, beta subunit	1.08	0.025914
1416278_a_at	ATP synthase, H+ transporting, mitochondrial F1 complex, O subunit	1.08	0.033193
1423737_at	NADH dehydrogenase (ubiquinone) Fe-S protein 3	1.07	0.175944
1448322_a_at	cytochrome c oxidase subunit IV isoform 1	1.07	0.044859
1416547_at	NADH dehydrogenase (ubiquinone) 1 beta subcomplex 3	1.06	0.131879
1428179_at	NADH dehydrogenase (ubiquinone) flavoprotein 2	1.06	0.112824
1426742_at	ATP synthase, H+ transporting, mitochondrial F0 complex, subunit b, isoform 1	1.06	0.137426
1416020_a_at	ATP synthase, H+ transporting, mitochondrial F0 complex, subunit c (subunit 9), isoform 1	1.06	0.178726
1448203_at	ATP synthase, H+ transporting, mitochondrial F0 complex, subunit g	1.06	0.161229
1422241_a_at	NADH dehydrogenase (ubiquinone) 1 alpha subcomplex, 1	1.05	0.197801
1418005_at	succinate dehydrogenase complex, subunit B, iron sulfur (Ip)	1.05	0.245014
1449218_at	cytochrome c oxidase, subunit VIIIb	1.05	0.168102
1454661_at	ATP synthase, H+ transporting, mitochondrial F0 complex, subunit c (subunit 9), isoform 3	1.05	0.186377
1423111_at	ATP synthase, H+ transporting, mitochondrial F1 complex, alpha subunit, isoform 1	1.05	0.171001
1452184_at	NADH dehydrogenase (ubiquinone) 1 beta subcomplex, 9	1.04	0.206517
1448483_a_at	NADH dehydrogenase (ubiquinone) 1 beta subcomplex, 2	1.03	0.570839
1416058_s_at	ATP synthase, H+ transporting, mitochondrial F1 complex, gamma polypeptide 1	1.03	0.354134
1422525_at	ATP synthase, H+ transporting, mitochondrial F1F0 complex, subunit e	1.03	0.494998
1432264_x_at	cytochrome c oxidase subunit VIIa polypeptide 2-like	1.02	0.514227
1416565_at	cytochrome c oxidase, subunit VIb	1.02	0.549193
1448112_at	cytochrome c oxidase, subunit VIIc	1.02	0.64915
1415980_at	ATP synthase, H+ transporting, mitochondrial F0 complex, subunit c (subunit 9), isoform 2	1.02	0.597775
1448589_at	NADH dehydrogenase (ubiquinone) 1 beta subcomplex, 5	1.01	0.801872
1435613_x_at	cytochrome c oxidase, subunit Vb	1.01	0.765462
1417607_at	cytochrome c oxidase, subunit VI a, polypeptide 2	1.01	0.700342
1422483_a_at	cytochrome c, somatic	1.01	0.689682
1448934_at	NADH dehydrogenase (ubiquinone) 1 alpha subcomplex 10	1	0.988797
1424085_at	NADH dehydrogenase (ubiquinone) 1 alpha subcomplex, 4	1	0.992668
1448427_at	NADH dehydrogenase (ubiquinone) 1 alpha subcomplex, 6 (B14)	1	0.994966
1415970_at	cytochrome c oxidase, subunit VIc	1	0.891803
1416143_at	ATP synthase, H+ transporting, mitochondrial F0 complex, subunit F	-1	0.97226
1416567_s_at	ATP synthase, H+ transporting, mitochondrial F1 complex, epsilon subunit	-1	0.905977
1429708_at	NADH dehydrogenase (ubiquinone) 1 alpha subcomplex	-1.01	0.861906
1428075_at	NADH dehydrogenase (ubiquinone) 1 beta subcomplex 4	-1.02	0.424323
1448198_a_at	NADH dehydrogenase (ubiquinone) 1 beta subcomplex 8	-1.02	0.51054
1417368_s_at	NADH dehydrogenase (ubiquinone) 1 alpha subcomplex, 2	-1.03	0.325652
1455997_a_at	ubiquinol-cytochrome c reductase binding protein	-1.03	0.407613
1416285_at	NADH dehydrogenase (ubiquinone) 1, subcomplex unknown, 1	-1.04	0.231232
1416494_at	NADH dehydrogenase (ubiquinone) Fe-S protein 5	-1.06	0.234851
1428464_at	NADH dehydrogenase (ubiquinone) 1 alpha subcomplex, 3	-1.07	0.207324
1428360_x_at	NADH dehydrogenase (ubiquinone) 1 alpha subcomplex, 7 (B14.5a)	-1.07	0.404494

B.

Expression of fatty acid metabolism genes

Fatty acid oxidation

		Fold Change	p-value
1436756_x_at	L-3-hydroxyacyl-Coenzyme A dehydrogenase, short chain	3.17	0.035803
1422526_at	fatty acid Coenzyme A ligase, long chain 2	1.94	0.000001
1423109_s_at	solute carrier family 25 (mitochondrial carnitine/acylcarnitine translocase), member 20	1.8	0.000031
1422811_at	solute carrier family 27 (fatty acid transporter), member 1	1.72	0.00048
1448987_at	acetyl-Coenzyme A dehydrogenase, long-chain	1.72	0.000025
1428146_s_at	acetyl-Coenzyme A acyltransferase 2 (mitochondrial 3-oxoacyl-Coenzyme A thiolase)	1.71	0.000003
1416772_at	carnitine palmitoyltransferase 2	1.62	0.000383
1452173_at	hydroxyacyl-Coenzyme A dehydrogenase/3-ketoacyl-Coenzyme A thiolase/enoyl-Coenzyme A hydratase (trifunctional protein), alpha subunit	1.59	0.000186
1419367_at	2,4-dienoyl CoA reductase 1, mitochondrial	1.49	0.000165
1452341_at	enoyl Coenzyme A hydratase, short chain, 1, mitochondrial	1.46	0.000009
1424184_at	acyl-Coenzyme A dehydrogenase, very long chain	1.43	0.000005
1429581_at	acyl-Coenzyme A dehydrogenase family, member 9	1.4	0.051528
1418321_at	dodecenoyl-Coenzyme A delta isomerase (3,2 trans-enoyl-Coenyme A isomerase)	1.39	0.000143
1426522_at	hydroxyacyl-Coenzyme A dehydrogenase/3-ketoacyl-Coenzyme A thiolase/enoyl-Coenzyme A hydratase (trifunctional protein), beta subunit	1.39	0.000027
1415984_at	acetyl-Coenzyme A dehydrogenase, medium chain	1.36	0.000015
1460216_at	acyl-Coenzyme A dehydrogenase, short chain	1.34	0.005249
1428082_at	fatty acid Coenzyme A ligase, long chain 5	1.3	0.179453
1422820_at	lipase, hormone sensitive	1.25	0.07633
1448382_at	enoyl-Coenzyme A, hydratase/3-hydroxyacyl Coenzyme A dehydrogenase	1.25	0.094294
1451828_a_at	fatty acid-Coenzyme A ligase, long chain 4	1.22	0.232356
1455446_x_at	acyl-Coenzyme A dehydrogenaseshort/branched chain	1.2	0.023054
1419262_at	acyl-Coenzyme A dehydrogenase family, member 8	1.18	0.009782
1417008_at	carnitine acetyltransferase	1.11	0.006071
1438391_x_at	hydroxyacyl-Coenzyme A dehydrogenase type II	-1.01	0.94904
1424441_at	solute carrier family 27 (fatty acid transporter), member 4	-1.06	0.491068
1434866_x_at	carnitine palmitoyltransferase 1a, liver	-1.06	0.871143
1452771_s_at	fatty acid Coenzyme A ligase, long chain 3	-1.39	0.04601

Fatty acid synthesis

1427052_at	acetyl-Coenzyme A carboxylase beta	1.21	0.000589
1423828_at	fatty acid synthase	1.04	0.914406
1451666_at	ATP citrate lyase	1.17	0.669932
1428190_at	solute carrier family 25 (mitochondrial carrier; citrate transporter), member 1	-1.02	0.893592
1427595_at	Mus musculus transcribed sequence with strong similarity to protein prf:2111499A (H.sapiens) 2111499A Ac-CoA carboxylase [Homo sapiens]	1.01	0.963376

Triglyceride synthesis

1423439_at	phosphoenolpyruvate carboxykinase 1, cytosolic	3.82	0.028271
1422678_at	diacylglycerol O-acyltransferase 2	1.82	0.002742
1425834_a_at	glycerol-3-phosphate acyltransferase, mitochondrial	1.17	0.215911
1418295_s_at	diacylglycerol O-acyltransferase 1	1.02	0.887869
1422703_at	glycerol kinase	-1.00	0.993038
1421025_at	1-acylglycerol-3-phosphate O-acyltransferase 1 (lysophosphatidic acid acyltransferase, alpha)	-1.22	0.026126
1416204_at	glycerol-3-phosphate dehydrogenase 1 (soluble)	-1.28	0.065895
1428323_at	glycerol phosphate dehydrogenase 2, mitochondrial	-1.43	0.002494
1416383_a_at	pyruvate carboxylase	-1.78	0.00045

C.

Expression of glycolysis and TCA cycle genes

glycolysis

		Fold Change	p-value
1416183_a_at	lactate dehydrogenase 2, B chain	1.78	0.000591
1426554_a_at	phosphoglycerate mutase 1	1.33	0.054072
1450269_a_at	phosphofructokinase, liver, B-type	1.29	0.067309
1422612_at	hexokinase 2	1.1	0.049277
1434773_a_at	solute carrier family 2 (facilitated glucose transporter), member 1	1.1	0.432057
1415958_at	solute carrier family 2 (facilitated glucose transporter), member 4	1.01	0.840103
1427404_x_at	enolase 1, alpha non-neuron	1	0.912503
1433604_x_at	aldolase 1, A isoform	-1.01	0.628201
1429972_s_at	thioredoxin reductase 2	-1.02	0.892226
1450081_x_at	glucose phosphate isomerase 1	-1.04	0.413958
1423869_s_at	thioredoxin reductase 3	-1.04	0.744204
1417951_at	enolase 3, beta muscle	-1.05	0.09062
1438640_x_at	gb:AV305101 /DB_XREF=gi:6337615 /DB_XREF=AV305101 =phosphoglycerate kinase 1	-1.05	0.230747
1417308_at	pyruvate kinase, muscle	-1.06	0.131154
1416780_at	phosphofructokinase, muscle	-1.09	0.05325
1435659_a_at	gene rich cluster, C9 gene	-1.14	0.003632

TCA cycle

1427441_a_at	succinate-Coenzyme A ligase, GDP-forming, beta subunit	1.54	0.000007
1450048_a_at	vacuolar protein sorting 33B (yeast)	1.46	0.023674
1418885_a_at	isocitrate dehydrogenase 3 (NAD+) beta	1.38	0.037347
1422500_at	isocitrate dehydrogenase 3 (NAD+) alpha	1.32	0.000638
1422433_s_at	isocitrate dehydrogenase 1 (NADP+), soluble	1.25	0.080736
1422577_at	citrate synthase	1.22	0.004511
1426688_at	succinate dehydrogenase complex, subunit A, flavoprotein (Fp)	1.22	0.000935
1416789_at	isocitrate dehydrogenase 3 (NAD+), gamma	1.19	0.00045
1449137_at	pyruvate dehydrogenase E1 alpha 1	1.18	0.025685
1427153_at	branched chain ketoacid dehydrogenase E1, beta polypeptide	1.16	0.08756
1452206_at	succinate-Coenzyme A ligase, ADP-forming, beta subunit	1.16	0.017195
1428235_at	succinate dehydrogenase complex, subunit D, integral membrane protein	1.14	0.008051
1415891_at	succinate-CoA ligase, GDP-forming, alpha subunit	1.14	0.00574
1416090_at	pyruvate dehydrogenase (lipoamide) beta	1.14	0.035142
1448630_a_at	succinate dehydrogenase complex, subunit C, integral membrane protein	1.12	0.003165
1424828_a_at	fumarate hydratase 1	1.11	0.038844
1456090_at	pyruvate dehydrogenase complex, component X	1.11	0.50992
1451002_at	gb:AA034553 /DB_XREF=gi:1506544 /DB_XREF=mi52a07.r1 / gb:NM_080633.1	1.09	0.062283
1418005_at	succinate dehydrogenase complex, subunit B, iron sulfur (Ip)	1.05	0.245014
1448172_at	malate dehydrogenase, soluble	1.04	0.430121
1416478_a_at	malate dehydrogenase, mitochondrial	1.03	0.382213

Affymetrix microarray analysis of mRNA levels in wt vs null gastrocnemius muscle in adult mice fed a normal chow diet (*n*=6).
A. Expression of OXPHOS pathway genes.
B. Expression of fatty acid metabolism genes
C. Expression analysis of glycolysis and TCA cycle genes

Table S4. PPARδ target genes upregulated by the absence of RIP140

Sequence ID	Primary Sequence Name		fold change	p-value
1416023_at	Fabp3	fatty acid binding protein 3, muscle and heart	2.14	0
1418472_at	Aspa	aspartoacylase (aminoacylase) 2	2.05	0.001477
1460316_at	Acsl1	fatty acid Coenzyme A ligase, long chain 2	1.63	0.000072
1455061_a_at	Acaa2	acetyl-Coenzyme A acyltransferase 2 (mitochondrial 3-oxoacyl-Coenzyme A thiolase)	1.94	0.00001
1417273_at	Pdk4	pyruvate dehydrogenase kinase, isoenzyme 4	1.69	0.009131
1447820_x_at	Cpt2	carnitine palmitoyltransferase 2	1.85	0.006656
1448987_at	Acadl	acetyl-Coenzyme A dehydrogenase, long-chain	1.72	0.000025
1452173_at	Hadha	hydroxyacyl-Coenzyme A dehydrogenase/3-ketoacyl-Coenzyme A thiolase	1.59	0.000186
1418328_at	Cpt1b	carnitine palmitoyltransferase 1, muscle	1.35	0.000124
1448188_at	Ucp2	uncoupling protein 2, mitochondrial	1.5	0.056108

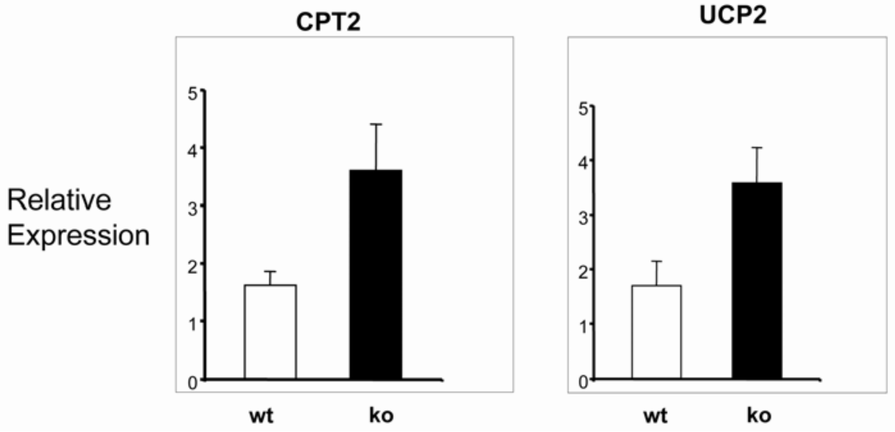

Affymetrix microarray and real-time PCR analysis of mRNA levels in wt vs null gastrocnemius muscle in adult mice fed a normal chow diet (*n*=6). PPAR δ target genes as referenced in (Dressel et al. 2003; Tanaka et al. 2003; Hummasti and Tontonoz. 2006).

Table S5. ERRα target genes upregulated by the absence of RIP140

1417956_at	Cidea	cell death-inducing DNA fragmentation factor, alpha subunit-like effector A	11.76	0.002751
1423439_at	Pepck	phosphoenolpyruvate carboxykinase 1, cytosolic	3.82	0.028271
1423860_at	Ptgds	prostaglandin D2 synthase (brain)	3.62	0.01569
1439675_at	Ppara	peroxisome proliferator activated receptor alpha	2.03	0.001399
1415984_at	Mcad	acetyl-Coenzyme A dehydrogenase, medium chain	1.36	0.000015
1460652_at	Erra	estrogen related receptor, alpha	1.36	0.000365

Affymetrix microarray analysis of mRNA levels in wt vs null gastrocnemius muscle in adult mice fed a normal chow diet (*n*=6). ERRα target genes as identified and referenced in (Luo et al. 2003; Huss et al. 2004; Herzog et al. 2006)

Table S6. Expression profile of early and late myogenic markers

1420757_at	myogenic factor 5	-1.18	0.700905
1426731_at	desmin	-1.02	0.750117
1417614_at	creatine kinase, muscle	1.01	0.646435
1419391_at	myogenin	1.36	0.115791

Affymetrix microarray analysis of mRNA levels in wt vs null gastrocnemius muscle in adult mice fed a normal chow diet (*n*=6).

Supplemental References

Dressel, U., Allen, T.L., Pippal, J.B., Rohde, P.R., Lau, P., and Muscat, G.E. 2003/12. The peroxisome proliferator-activated receptor beta/delta agonist, GW501516, regulates the expression of genes involved in lipid catabolism and energy uncoupling in skeletal muscle cells. *MolEndocrinol* 17(12): 2477-2493.

Herzog, B., Cardenas, J., Hall, R.K., Villena, J.A., Budge, P.J., Giguere, V., Granner, D.K., and Kralli, A. 2006. Estrogen-related receptor alpha is a repressor of phosphoenolpyruvate carboxykinase gene transcription. *J Biol Chem* 281(1): 99-106.

Hummasti, S. and Tontonoz, P. 2006. The peroxisome proliferator-activated receptor N-terminal domain controls isotype-selective gene expression and adipogenesis. *Mol Endocrinol* 20(6): 1261-1275.

Huss, J.M., Torra, I.P., Staels, B., Giguere, V., and Kelly, D.P. 2004/10. Estrogen-related receptor alpha directs peroxisome proliferator-activated receptor alpha signaling in the transcriptional control of energy metabolism in cardiac and skeletal muscle. *MolCell Biol* 24(20): 9079-9091.

Luo, J., Sladek, R., Carrier, J., Bader, J.A., Richard, D., and Giguere, V. 2003. Reduced fat mass in mice lacking orphan nuclear receptor estrogen-related receptor alpha. *Mol Cell Biol* 23(22): 7947-7956.

Tanaka, T., Yamamoto, J., Iwasaki, S., Asaba, H., Hamura, H., Ikeda, Y., Watanabe, M., Magoori, K., Ioka, R.X., Tachibana, K., Watanabe, Y., Uchiyama, Y., Sumi, K., Iguchi, H., Ito, S., Doi, T., Hamakubo, T., Naito, M., Auwerx, J., Yanagisawa, M., Kodama, T., and Sakai, J. 2003/12/23. Activation of peroxisome proliferator-activated receptor delta induces fatty acid beta-oxidation in skeletal muscle and attenuates metabolic syndrome. *ProcNatlAcadSciUSA* 100(26): 15924-15929.
